# Adverse childhood experiences, DNA methylation age acceleration, and cortisol in UK children: a prospective population-based cohort study

**DOI:** 10.1186/s13148-020-00844-2

**Published:** 2020-04-07

**Authors:** Rosalind Tang, Laura D. Howe, Matthew Suderman, Caroline L. Relton, Andrew A. Crawford, Lotte C. Houtepen

**Affiliations:** 1 Bristol Medical School, Faculty of Health Sciences, University of Bristol, 5 Tyndall Avenue, Bristol, BS8 1UD UK; 2 Keenan Research Centre for Biomedical Science, St. Michael’s Hospital, Toronto, Canada; 3 MRC Integrative Epidemiology Unit, University of Bristol, Bristol, UK; 4 BHF Centre for Cardiovascular Science, The Queen’s Medical Research Institute, University of Edinburgh, Edinburgh, UK

**Keywords:** Adversity, Adverse childhood experiences, Child abuse, DNA methylation, Emotional abuse, Epigenetics, Longitudinal studies

## Abstract

**Background:**

Epigenetic mechanisms may partly explain the persistent effects of adverse childhood experiences (ACEs) on health outcomes in later life. DNA methylation can predict chronological age, and advanced methylation-predicted age beyond chronological age (DNA methylation age acceleration) is associated with ACEs, adverse mental and physical health, and elevated diurnal and baseline salivary cortisol. Childhood adversity is also associated with dysregulation of the hypothalamic-pituitary-adrenal axis, which produces the neuroendocrine hormone cortisol. It remains unknown whether these associations are specific to certain types of adversity. Herein, we investigate the associations of ACEs with DNA methylation age acceleration and plasma cortisol in the Avon Longitudinal Study of Parents and Children (ALSPAC) birth cohort.

**Methods:**

In this study of the children in ALSPAC, we used multiple linear regression to examine associations of cumulative exposure to ACE, as well as exposure to ten individual types of ACEs, with Horvath-estimated DNA methylation age acceleration and with baseline plasma cortisol. The ten ACEs were those included in the World Health Organization’s ACE International Questionnaire. Data on ACEs were prospectively collected from age 0–14 years. DNA methylation age acceleration and plasma cortisol were measured at mean 17.1 years and 15.5 years, respectively.

**Results:**

We included 974 UK children in the present study. Exposure to four or more ACEs compared to zero was associated with DNA methylation age acceleration in girls (*β*, 95% CI = 1.65, 0.25 to 3.04 years) but not in boys (*β*, 95% CI = − 0.11, − 1.48 to 1.26 years). Also, in girls, emotional abuse and physical abuse were each associated with DNA methylation age acceleration (*β*, 95% CI = 1.20, 0.15 to 2.26 years and *β*, 95% CI = 1.22, 0.06 to 2.38 years, respectively). No other ACEs were associated with accelerated DNA methylation age in either sex. Associations were also null between ACE and cortisol, and cortisol and DNA methylation age acceleration.

**Conclusions:**

In this prospective population-based study of UK children, cumulative ACE exposure, emotional abuse, and physical abuse between age 0 and 14 years were each associated with Horvath-estimated DNA methylation age acceleration at age 17 years in girls but not in boys.

## Background

While there is a large body of evidence documenting the long-term consequences of adverse childhood experiences (ACEs) on social and health outcomes in later life [1, 2], the mechanisms through which they occur remain unclear. Epigenetic mechanisms may help to explain the lasting effects of early life adversity [3]. “Epigenetic clocks,” which are sets of DNA methylation (DNAm) markers (CpG sites) that accurately predict chronological aging, have been recently described [4–6], and higher DNAm-predicted age relative to chronological age [DNAm age acceleration (AA)] is associated with cardiovascular disease [7], cancer [8], lower verbal fluency [9], and all-cause mortality [10]. DNAm AA has also been associated with childhood exposure to adversity, including parental depression [11], violence [12, 13], sexual abuse [14], low socioeconomic status [15–17], and cumulative exposure to sexual abuse, physical abuse, or neglect [18].

Dysregulation of the hypothalamic-pituitary-adrenal (HPA) axis is a potential mediator between childhood adversity, epigenetics, and poor health in later life. Researchers have reported altered HPA axis function and altered cortisol stress response in adult survivors of childhood abuse, trauma, and neglect [19]. DNAm AA is also associated with elevated diurnal [20] and baseline [21] salivary cortisol in adolescents. Here, we investigate the associations of individual types of ACEs, as well as cumulative ACE exposure, with DNAm AA and plasma cortisol in the Avon Longitudinal Study of Parents and Children (ALSPAC) birth cohort.

## Results

**Fig. 1:**
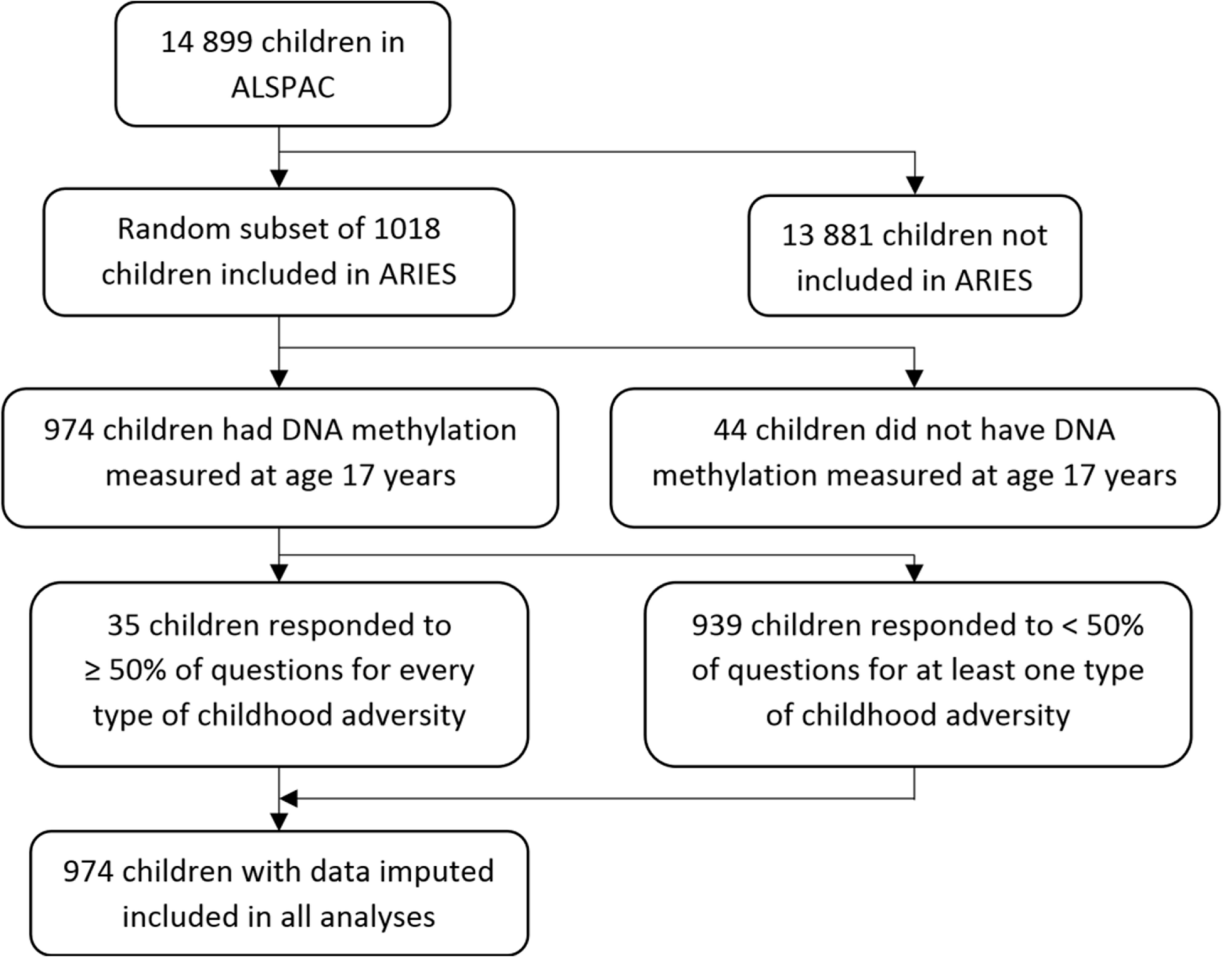
Flowchart of study population. ALSPAC, Avon Longitudinal Study of Parents and Children; ARIES, Accessible Resource for Integrated Epigenomic Studies

In ALSPAC, 974 children had DNAm measured at age 17 years, and we included these children in all analyses (Fig. 1). Baseline characteristics are reported in Table 1. A comparison of these children and the rest of the ALSPAC cohort is reported in Additional file 1: Table S1.

**Table 1:** Characteristics of the study population

Characteristics	Mean (SE) or % in girls	Mean (SE) or % in boys	*P* value^a^
*N*	500	474	
Chronological age	17.11 (0.05)	17.16 (0.05)	0.46
DNA methylation age acceleration (years)	− 0.24 (0.18)	0.25 (0.17)	0.05
Plasma cortisol (nmol/L)	522.54 (15.99)	465.85 (11.24)	< 0.01
Count of adverse childhood experiences			0.85
None	21.5	22.0	
One	24.4	25.9	
Two or three	35.9	32.7	
Four or more	18.2	19.4	
Individual adverse childhood experience exposure			
Bullying	14.4	17.1	0.26
Emotional abuse	22.9	21.9	0.75
Emotional neglect	14.6	16.9	0.34
Parent mental health problem	52.7	47.2	0.13
Parent convicted	13.1	11.6	0.60
Parental separation	20.9	25.9	0.11
Physical abuse	18.0	17.8	0.95
Sexual abuse	4.9	2.0	0.05
Household substance abuse	13.4	13.9	0.85
Violence in household	21.3	21.9	0.85

*SE* standard error

^a^Two-tailed *P* value

**Fig. 2:**
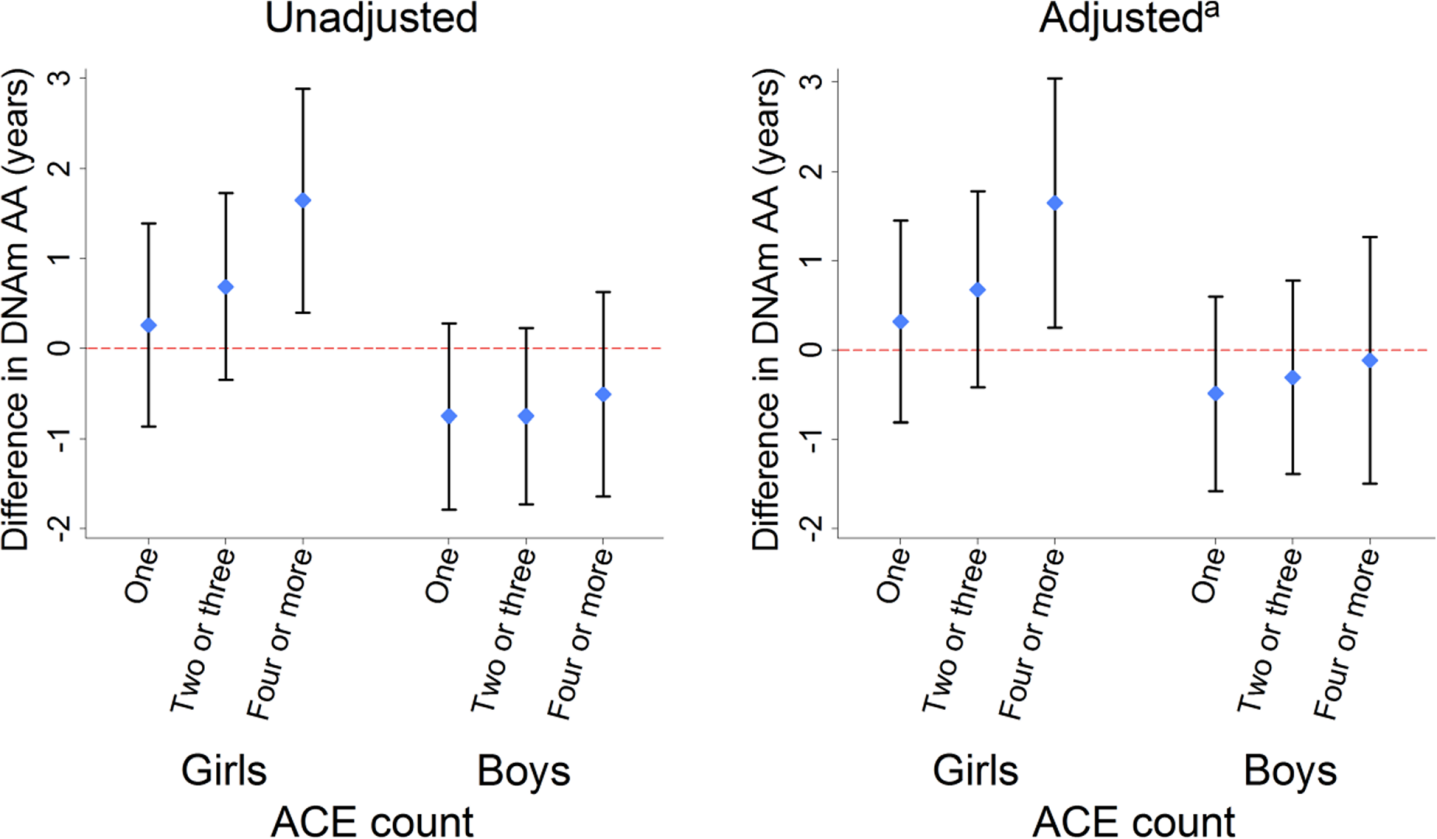
Cumulative exposure to adverse childhood experiences from age 0–14 years and DNA methylation age acceleration at age 17 years. DNAm AA, Horvath-estimated DNA methylation age acceleration; ACE, adverse childhood experiences. Scatter plots show mean differences in DNAm AA for each category of ACE exposure when compared to zero exposure to ACE. Whiskers show 95% confidence intervals. ^a^Adjusted for white blood cell composition, smoking status, maternal body mass index, maternal smoking, maternal age at delivery, maternal depression, partner’s depression, mother’s highest education qualification, household’s highest socioeconomic class

We found no evidence of association between ACE and DNAm AA when analyzing boys and girls together (Additional file 1: Table S2). However, we observed sex interactions in all regression models (Additional file 1: Table S2 and Additional file 1: Table S3). Table 2 shows the sex-stratified associations of ACE and DNAm AA. Exposure to four or more ACEs compared to zero was associated with DNAm AA in girls (Table 2 and Fig. 2). The adjusted mean differences in DNAm AA (95% confidence interval (CI)) among girls were 0.32 (− 0.81, 1.45) years for an ACE count of one, 0.68 (− 0.42, 1.78) years for an ACE count of two or three, and 1.65 (0.25, 3.04) years for an ACE count of four or more when compared to zero. In our analyses of individual types of adversity, emotional abuse and physical abuse were each associated with DNAm AA in girls (Table 2). The adjusted mean differences in DNAm AA (95% CI) were 1.20 (0.15, 2.26) years for emotional abuse, and 1.22 (0.06, 2.38) years for physical abuse when compared to the absence of emotional or physical abuse, respectively. We found no evidence of association between DNAm AA and ACE count, or any individual ACE, in boys (Table 2 and Fig. 2). No associations were consistent between Horvath (Table 2) and Hannum (Additional file 1: Table S4) DNAm AA measures.

**Table 2:** Adverse childhood experience and DNA methylation age acceleration

Exposure	Unadjusted mean difference in DNA methylation age acceleration, years (± 95% CI)		Adjusted^a^ mean difference in DNA methylation age acceleration, years (± 95% CI)	
	Girls	Boys	Girls	Boys
Count of ACEs				
None	0	0	0	0
One	0.26 (− 0.86, 1.39)	− 0.75 (− 1.79, 0.28)	0.32 (− 0.81, 1.45)	− 0.48 (− 1.56, 0.60)
Two or three	0.69 (− 0.35, 1.73)	− 0.75 (− 1.73, 0.23)	0.68 (− 0.42, 1.78)	− 0.30 (− 1.37, 0.78)
Four or more	1.65 (0.40, 2.89)^b^	− 0.51 (− 1.64, 0.63)	1.65 (0.25, 3.04)^b^	− 0.11 (− 1.48, 1.26)
Individual ACE exposure				
Bullying	0.42 (− 0.63, 1.47)	− 0.77 (− 1.70, 0.16)	0.30 (− 0.79, 1.39)	− 0.61 (− 1.63, 0.40)
Emotional abuse	0.96 (− 0.01, 1.94)	− 0.49 (− 1.40, 0.42)	1.20 (0.15, 2.26)^b^	− 0.31 (− 1.34, 0.71)
Emotional neglect	− 0.23 (− 1.24, 0.79)	0.77 (− 0.14, 1.68)	− 0.45 (− 1.53, 0.64)	0.31 (− 0.69, 1.30)
Parent mental health problem	0.92 (0.11, 1.73)^b^	− 0.60 (− 1.34, 0.13)	0.91 (− 0.01, 1.83)	− 0.21 (− 1.05, 0.64)
Parent convicted	0.95 (− 0.40, 2.30)	− 0.12 (− 1.37, 1.13)	0.56 (− 1.00, 2.12)	− 0.15 (− 1.51, 1.21)
Parental separation	0.85 (− 0.15, 1.86)	0.19 (− 0.63, 1.02)	0.90 (− 0.29, 2.10)	0.62 (− 0.34, 1.58)
Physical abuse	1.29 (0.18, 2.41)^b^	− 0.05 (− 1.07, 0.97)	1.22 (0.06, 2.38)^b^	0.04 (− 1.11, 1.19)
Sexual abuse	1.04 (− 0.84, 2.92)	− 0.48 (− 3.17, 2.20)	1.29 (− 0.71, 3.30)	− 0.75 (− 3.67, 2.17)
Household substance abuse	0.82 (− 0.52, 2.16)	0.36 (− 0.71, 1.44)	0.72 (− 0.78, 2.22)	0.43 (− 0.78, 1.65)
Violence in household	0.19 (− 0.82, 1.20)	0.26 (− 0.69, 1.20)	− 0.15 (− 1.28, 0.99)	0.72 (− 0.35, 1.79)

*95% CI* 95% confidence interval, *ACE* adverse childhood experience

^a^Adjusted for white blood cell composition, smoking status, maternal body mass index, maternal smoking, maternal age at delivery, maternal depression, partner’s depression, mother’s highest education qualification, household’s highest socioeconomic class

^b^Two-tailed *P* value < 0.05

We found no evidence of associations between any ACE measures and morning plasma cortisol (Additional file 1: Table S5). Associations between DNAm AA with morning plasma cortisol were also null.

## Discussion

In this prospective study of 974 UK children, exposure to four or more ACEs compared to zero was associated with positive Horvath DNAm AA in girls only. Also in girls, emotional abuse and physical abuse were each associated with positive Horvath DNAm AA. This suggests that exposure to certain types of childhood adversity is related to accelerated DNA methylation aging in girls.

Two previous studies examined the association between cumulative exposure to childhood adversity and Horvath-estimated DNAm AA [13, 18]. In a meta-analysis, Wolf et al. [18] did not find evidence of an association between cumulative ACE exposure measured using the Childhood Trauma Questionnaire (sexual abuse, physical abuse, and physical neglect items only), nor the Traumatic Life Events Questionnaire (childhood items only), with Horvath DNAm AA. What may contribute to this difference in findings is the exposure measure. Wolf et al. [18] used retrospective self-administered questionnaires, thus relying on one source and reporting at a single time point, whereas we used a variety of measures reported by the child and mother at multiple time points.

The other study investigating cumulative exposure to childhood adversity and Horvath DNAm AA [13] did report an association between DNAm AA and cumulative childhood exposure to threat, but not exposure to deprivation. The threat metric used by Sumner et al. [13] included emotional abuse, sexual abuse, physical abuse, domestic violence, and interpersonal violence, while their deprivation metric included emotional neglect, physical neglect, food insecurity, and/or cognitive deprivation. Thus, the threat metric includes both emotional abuse and physical abuse, the two ACEs that appear to drive the association between our cumulative ACE measure and Horvath DNAm AA in girls. Interestingly, no previous study performed sex-stratified analyses, despite Sumner et al. [13] identifying a sex difference in DNAm AA. Numerous other studies also describe sex differences in DNAm AA [5, 11, 13, 18, 22–24]. Other key differences between the two previous studies and the present study include examining individual types of adversity, prospective reporting of ACE exposure, and data reporting from multiple sources.

Several reports have examined the association between Horvath DNAm AA and individual types of ACEs [11–18, 25], although few studies use prospectively collected ACE data [11, 25]. Brody et al. [11] reported a positive association between primary caregivers’ (91.5% mothers’) self-reported depressive symptoms when their children were age 11 years and the children’s Horvath DNAm AA at age 20 years. Using a cutoff score of 16 on the Center for Epidemiologic Studies Depression Scale, Brody et al. [11] categorized caregivers as having higher versus lower depressive symptoms. In our study, we did not look specifically at parental depressive symptoms, but instead, we examined an association between DNAm AA and a broader measure of parental mental health that also includes self-harm, attempted suicide, medication for depression, schizophrenia, eating disorders, medication for anxiety, and hospital admission for any type of mental health issue. We did not find evidence of an association between this broader measure of mental health and DNAm AA. Another study using ALSPAC data examined the association between ACE exposures up to age 7 years and DNAm AA at age 7 years [25]. Marini et al. [25] reported null associations between Horvath DNAm AA and physical or emotional abuse, physical or sexual abuse, maternal psychopathology, and one adult in the household at this younger age. Our exposures differed from those of Marini et al. [25], as they did not investigate physical, emotional, and sexual abuse individually, nor did they investigate cumulative exposure to more than one type of adversity. In the present study, we included seven additional years of ACE data beyond Marini et al. [25], with a subsequently higher burden of childhood adversity, and we were able to investigate all ten types of ACE included in the World Health Organization (WHO) ACE International Questionnaire [26]. Studies using retrospectively self-reported ACE data have estimated positive associations between Horvath DNAm AA and childhood exposure to violence measured using the Violence Exposure Scale for Children-Revised [12, 13], as well as childhood exposure to sexual abuse [14], where we did not find evidence of such associations in the children of ALSPAC.

Overall, we found greater DNAm AA in boys relative to girls, which is consistent with most reports [18], though not all [13]. The differential associations we estimated between ACE and DNAm AA between sexes are also consistent with previous reports of sex differences in the DNAm sites associated with both childhood adversity and adult body mass index [27] and consistent with reports of stronger associations between ACE and adverse mental health in adult women compared to adult men [28]. Sex differences in the effect of ACE on DNAm age may help to explain these lifelong differences.

We set out to examine the bidirectional effects of cortisol and DNAm AA following childhood adversity, but we did not pursue this line of enquiry because we found no evidence of association between ACE and baseline plasma cortisol at age 15 years. The cortisol system is highly complex and dynamic; therefore, further research using other measures for cortisol, such as a dynamic measure of cortisol, is needed to further examine the role of cortisol in the relationship between childhood adversity and DNAm AA.

Strengths of this study include the prospective design and reporting of childhood adversity from multiple sources. Due to the sensitive nature of the ACEs, under-reporting remains likely and there is a high proportion of missing data in our non-imputed dataset. This likely contributed to the higher estimates of ACE exposure in our imputed data compared to our non-imputed data (Additional file 1: Tables S6-S8). However, our imputed estimates of ACE count, as well as individual types of ACE, were similar to those reported in other population-based birth cohorts including the UK Environmental Risk Longitudinal Twin Study [29], the Growing Up in Scotland Birth Cohort 1 [30], the New Zealand Dunedin cohort [31], the Australian Temperament Project [32], and the most recent birth cohort in the CDC-Kaiser ACE Study [33]. The one exception was exposure to parent mental health problem (52.7% in girls and 47.2% in boys), which was higher compared to most other studies of ACEs although still in line with lifetime mental health prevalence estimates in the USA [34] and in Northern Ireland [35]. The high proportion of missing data in ALSPAC arises due to the derivation of ACE measures using 485 separate questionnaire items spanning 14 years of data collection from multiple respondents. While only 35 participants met our definition of a complete case (i.e., having ≥ 50% complete data for every ACE type), the majority of participants did contribute a significant amount of data to the imputation models. Previous research using these data has also confirmed expected and established relationships between exposure to ACE and outcomes such as depression, substance misuse, and educational attainment [36]. The Accessible Resource for Integrated Epigenomic Studies (ARIES) subset included any mother-child pairs in ALSPAC with maternal DNA samples available from two time points (antenatal clinic and follow-up clinic at child’s mean age 15.5 years) as well as child’s DNA samples available from three time points (birth, child’s mean age 7.5 years, and child’s mean age 15.5 years) [37]. This subset experienced greater cumulative exposure to ACE and greater individual exposure to emotional neglect or parental conviction when compared to the remaining children in ALSPAC (Additional file 1: Table S1). The children in ARIES were also more often White and more affluent, and we cannot exclude the effect of residual selection bias, although this would more likely lead to an under-estimate of ACE exposure.

## Conclusions

Cumulative ACE exposure and exposure to emotional abuse or physical abuse from age 0 to 14 years are positively associated with Horvath DNAm AA in girls but not boys at age 17 years. These findings suggest differential effects of childhood adversity on DNAm AA between sexes.

## Methods

### Participants

ALSPAC is a population-based cohort study which recruited 14,541 pregnant women with expected delivery dates from 1 April 1991 to 31 December 1992 in Avon, UK [38, 39]. A total of 14,899 children alive at 1 year were included [38, 39]. Data collection in the mothers, mothers’ partners, and children is ongoing via clinic visits, questionnaires, and data linkages to external organizations. The ALSPAC website contains details of all available data through a fully searchable data dictionary and variable search tool [40]. A sub-study of ALSPAC, the ARIES subset, was established to study epigenetics and health [37]. In ARIES, DNAm samples were collected in a subset of 1018 mother-child pairs. In the present study, we included any child in ARIES who had epigenome-wide DNAm measured at their adolescence clinic visit (mean age 17.1 years).

### Measures

Both ALSPAC participants themselves and their mothers reported on multiple forms of ACE at multiple time points. Full details of the derivation of ACE measures have been described elsewhere [41]. Briefly, dichotomous constructs indicating exposure to adversities between birth and 14 years were created for the ten ACEs that are included in the WHO ACE International Questionnaire [26]. These ten ACEs are bullying, emotional abuse, emotional neglect, parental mental illness or suicide attempt, parental criminal conviction, parental separation, physical abuse, sexual abuse, parental substance abuse, and violence between parents. How we define each type of adversity is described in Additional file 1: Table S9. Most ACE data were collected prospectively, but some (in particular, sexual abuse) included retrospective reports. The ACE data used in the present study (Table 1) differ from those reported in the data note by Houtepen et al. [41] because we used a smaller subset of the ALSPAC cohort; we defined our exposure period as age 0–14 years rather than 0–16 years because plasma cortisol was measured at target age 15.5 years, and our imputation model included additional covariates required for our regression analyses. Overall, the prevalence estimates in our imputed data were similar to that in the original data note [41].

Peripheral blood was collected at mean age 17.1 years in ARIES, and DNAm was quantified using Illumina Infinium HumanMethylation450K BeadChip assay (Illumina Inc., San Diego, CA) as previously described by Relton et al. [37]. DNAm AA was calculated as the residuals from regression of DNAm age on chronological age. We describe a positive DNAm age residual as DNAm AA and a negative residual as DNAm age deceleration. Most studies of ACE and DNAm AA use the Horvath, rather than Hannum, epigenetic clock [11–14, 17]. The Horvath method has been validated in different tissue types and in samples from children and adults [42], whereas the Hannum clock was developed using adult whole blood samples [5]. However, a recent meta-analysis by Wolf et al. [18] reported a positive association between cumulative ACE count and Hannum DNAm AA but null association with Horvath DNAm AA. Thus, for completeness, we examine ACE with Horvath estimates in our main analyses and with Hannum estimates in Additional file 1: Table S4.

Plasma cortisol was measured by radioimmunoassay in another subset of ALSPAC children with the target age of 15.5 years. Of the 9985 children invited, 5198 attended (response rate 52%). An additional 55 children attended after further outreach. Morning cortisol measurements were available in 1567 children though not all of these children were included in the present study, i.e., those who did not have DNAm quantified at age 17 years were not included (Fig. 1).

### Statistical analyses

The ACE measures were derived from multiple questionnaires and clinics over a long time period (birth to age 14 years). No participants had data on all of the individual questionnaire items (> 500 separate questions relating to ACE), necessitating the use of multivariate multiple imputation to avoid exclusion of participants, minimize selection bias, and account for missing data. We derived a dichotomous construct indicating presence or absence of each ACE if a participant responded to ≥ 50% of the questions related to a given ACE. If the participant responded to < 50% of the questions for a given ACE, we set the dichotomous indicator to missing. Of the 974 participants with DNA methylation measured at age 17 years, 35 met our definition of a complete case (≥ 50% of questions for each ACE). Variables included in our imputation model were those in our analyses (exposures, covariates, outcomes) or predictors of missingness. Predictors of missingness are auxiliary variables selected to inform the generation of unbiased estimates of missing values and to improve the missing at random assumption [43]. We included twelve auxiliary variables from the mothers’ and partners’ social history during pregnancy and nine auxiliary childhood adversity exposures not part of the WHO ACE International Questionnaire. The distribution of all variables in the imputation model is listed in Additional file 1: Table S10, and how they were included in the model are reported in Additional file 1: Table S11. Sex differences in DNAm age and AA have been described in ALSPAC [23] and other cohorts [5, 11, 13, 18, 22, 24]. To allow for sex-stratified analyses, imputation was performed separately in male and female participants, with imputed datasets subsequently appended. Additional file 1: Table S6 compares the distribution of variables in the non-imputed and imputed datasets. Additional file 1: Table S7 (girls) and Additional file 1: Table S8 (boys) separate these distributions by sex. The complete cases were more affluent than the full cohort, and a complete case analysis may have resulted in selection bias. Imputed estimates were combined using Rubin’s rules [44]. Likelihood-ratio tests for sex interaction were analyzed using Meng-Rubin’s rules [45].

We used multiple linear regression to examine associations of cumulative ACE count and exposure to individual ACEs with DNAm AA. Covariates were selected in a stepwise process, where potential confounders were individually added and subsequently removed if no effect on the model was observed. Models were analyzed unadjusted and then adjusted for white blood cell composition, smoking status at time of DNAm measurement, ethnicity, birth weight, gestational age at delivery, and maternal variables (smoking, pre-pregnancy weight, body mass index, home ownership status, age at delivery, parity, marital status, highest educational qualification, homelessness, Edinburgh Postnatal Depression Scale (EPDS) at 18 weeks and 32 weeks gestation, partner’s EPDS at 18 weeks gestation and household’s highest socioeconomic class at 18 weeks gestation). Body mass index may be a confounder or a mediator in the association between childhood adversity and DNAm AA [46]. To avoid over-adjustment, we excluded body mass index from our main analyses (Table 2), and the associations with further adjustment for body mass index are reported in Additional file 1: Table S12. The associations between exposure to childhood adversity and DNAm AA were similar with and without further adjustment for body mass index. Similar analyses using multiple linear regression were performed to investigate associations of ACE with cortisol, adjusting for cortisol sampling time, body mass index, and the same set of covariates as the DNAm AA analyses with the exclusion of white blood cell composition and child’s smoking status. Regression models were sex-stratified if an interaction was identified by likelihood-ratio test using Meng-Rubin’s rules [45]. All analyses were performed using Stata 15/MP (StataCorp, Austin, USA).

## Supplementary information

**Additional file 1:.** Supplementary tables.

## Data Availability

The data analyzed in the present study are available from ALSPAC: http://www.bristol.ac.uk/alspac/researchers/our-data/

## Abbreviations

ACE: Adverse childhood experience
ALSPAC: The Avon Longitudinal Study of Parents and Children
ARIES: The Accessible Resource for Integrated Epigenomic Studies
CI: Confidence interval
DNAm AA: DNA methylation age acceleration
EPDS: Edinburgh Postnatal Depression Scale
HPA axis: Hypothalamic-pituitary-adrenal axis
MRC: Medical Research Council
SE: Standard error
WHO: World Health Organization
