# Supplementary material for: Adverse childhood experiences, DNA methylation age acceleration, and cortisol in UK children: a prospective population-based cohort study
Source: Clin Epigenetics. 2020 Apr 7;12:55. doi: 10.1186/s13148-020-00844-2 (PMC7137290; doi:10.1186/s13148-020-00844-2)
Supplement: Supplementary file 1 — Additional file 1:. Supplementary tables. [file 13148_2020_844_MOESM1_ESM.docx]

**Adverse childhood experiences, DNA methylation age acceleration and cortisol in UK children: a prospective population-based cohort study**

**Authors:** Rosalind Tang^1,2,^*; Laura D. Howe^3^; Matthew Suderman^3^; Caroline L. Relton^3^; Andrew A. Crawford^3,4#^; Lotte C. Houtepen^3#^

**Affiliations:** ^1^Bristol Medical School, Faculty of Health Sciences, University of Bristol, United Kingdom; ^2^Keenan Research Centre for Biomedical Science, St. Michael’s Hospital, Toronto, Canada; ^3^MRC Integrative Epidemiology Unit, University of Bristol, United Kingdom; ^4^BHF Centre for Cardiovascular Science, The Queen's Medical Research Institute, University of Edinburgh, United Kingdom

^#^Joint last authors (these authors contributed equally)

# Additional file 1

### **Table S1.** Participants with and without DNA methylation data in ALSPAC

| **Variables** | **Mean (SE) or % in children without DNAm measured** | **Mean (SE) or % in children with DNAm measured** | ***P* value^a^** |
| --- | --- | --- | --- |
| N | 14 471 | 974 |  |
| Plasma cortisol (nmol/L) | 485.45 (4.43) | 485.79 (9.13) | 0.97 |
| Count of adverse childhood experiences |  |  |  |
| None | 79.3 | 40.8 | <0.001 |
| One | 6.5 | 18.4 |  |
| Two or three | 10.2 | 28.6 |  |
| Four or more | 4.0 | 12.2 |  |
| Individual adverse childhood experience exposures |  |  |  |
| Bullying | 6.4 | 14.5 | 0.65 |
| Emotional abuse | 9.4 | 18.3 | 0.92 |
| Emotional neglect | 8.7 | 17.1 | 0.003 |
| Parent mental health problem | 19.5 | 37.6 | 0.52 |
| Parent convicted | 16.6 | 40.3 | <0.001 |
| Parental separation | 9.7 | 17.2 | 0.26 |
| Physical abuse | 5.1 | 11.4 | 0.36 |
| Sexual abuse | 1.1 | 2.2 | 0.29 |
| Household substance abuse | 4.3 | 8.1 | 0.92 |
| Violence in household | 7.9 | 14.6 | 0.31 |
| **Covariates** | **Mean (SE) or % in children without DNAm measured** | **Mean (SE) or % in children with DNAm measured** | ***P* value^a^** |
| Cortisol sampling time from 08:00 (minutes) | 44.66 (0.56) | 43.83 (0.91) | 0.47 |
| Body mass index (kg/m^2^) at cortisol sampling | 21.47 (0.05) | 21.37 (0.11) | 0.43 |
| Maternal smoking during pregnancy |  |  | <0.001 |
| No | 51.5 | 78.2 |  |
| Early | 4.2 | 2.4 |  |
| Sustained | 16.2 | 10.4 |  |
| Child’s ethnicity is non-white | 4.1 | 2.7 | <0.001 |
| Child’s birth weight (g) | 3373.01 (5.15) | 3492.49 (15.85) | <0.001 |
| Child’s gestational age at delivery (weeks) | 38.32 (0.05) | 39.61 (0.05) | <0.001 |
| Maternal pre-pregnancy weight (kg) | 61.64 (0.11) | 61.61 (0.35) | 0.94 |
| Maternal body mass index during pregnancy (kg/m^2^) | 22.94 (0.04) | 22.78 (0.12) | 0.24 |
| Mother’s home ownership status during pregnancy |  |  | <0.001 |
| Mortgaged | 60.7 | 83.8 |  |
| Owned | 1.9 | 1.7 |  |
| Council rented | 13.0 | 5.7 |  |
| Rent (private and furnished) | 3.8 | 2.3 |  |
| Rent (private and unfurnished) | 2.7 | 1.2 |  |
| Housing authority rented | 1.5 | <0.5 |  |
| Other | 3.1 | 2.1 |  |
| Maternal age at delivery (years) | 27.88 (0.04) | 29.55 (0.14) | <0.001 |
| Maternal parity | 0.85 (0.01) | 0.73 (0.03) | <0.001 |
| Mother’s marital status during pregnancy |  |  | <0.001 |
| Never married | 17.2 | 11.9 |  |
| Widowed | 0.1 | <0.5 |  |
| Divorced | 3.8 | 3.6 |  |
| Separated | 1.4 | 1.1 |  |
| 1st marriage | 58.9 | 76.3 |  |
| Marriage 2 or 3 | 5.7 | 5.3 |  |
| Mother’s highest education qualification (self-reported) |  |  | <0.001 |
| Certificate of secondary education | 16.9 | 8.5 |  |
| Vocational | 8.0 | 7.2 |  |
| Ordinary level | 27.7 | 33.2 |  |
| Advanced level | 17.4 | 28.9 |  |
| Degree | 9.8 | 20.1 |  |
| Mother became homeless during pregnancy | 1.8 | 1.7 | 0.36 |
| Mother’s depression score (EPDS) at 18 weeks gestation | 7.04 (0.05) | 6.33 (0.15) | <0.001 |
| Mother’s depression score (EPDS) at 32 weeks gestation | 7.12 (0.05) | 6.56 (0.15) | 0.001 |
| Partner’s depression score (EPDS) at 18 weeks gestation | 4.24 (0.04) | 4.12 (0.14) | 0.40 |
| Household social class at 18 weeks gestation |  |  | <0.001 |
| I - Professional | 6.7 | 14.6 |  |
| II - Managerial and technical | 29.2 | 41.4 |  |
| IIINM - Skilled non-manual | 24.7 | 26.5 |  |
| IIIM - Skilled manual | 11.2 | 8.5 |  |
| IV - Partly skilled | 6.2 | 4.8 |  |
| V - Unskilled | 1.6 | 0.5 |  |
| **Auxiliary imputation variables** | **Mean (SE) or % in children without DNAm measured** | **Mean (SE) or % in children with DNAm measured** | ***P* value^a^** |
| Poor parent-child bonding | 7.9 | 14.4 | 0.17 |
| Financial difficulties | 6.5 | 9.4 | 0.015 |
| Intimate partner violence | 2.7 | 5.3 | 0.12 |
| Neighbourhood | 2.4 | 2.7 | 0.062 |
| Child physical illness | 2.9 | 4.5 | 0.12 |
| Parent physical illness | 2.8 | 8.3 | 0.53 |
| Low socioeconomic status | 4.8 | 7.1 | 0.073 |
| Child lacking social support | 4.2 | 9.7 | 0.87 |
| Parent lacking social support | 5.5 | 8.5 | 0.13 |
| Partner’s highest education qualification (mother-reported) |  |  | <0.001 |
| Certificate of secondary education | 20.7 | 14.8 |  |
| Vocational | 6.5 | 7.2 |  |
| Ordinary level | 16.2 | 21.1 |  |
| Advanced level | 19.7 | 28.0 |  |
| Degree | 13.4 | 25.4 |  |
| Partner became homeless during pregnancy | 0.8 | 0.5 | 0.11 |
| Difficulty in affording heating during pregnancy | 2.4 | 2.0 | 0.059 |
| Difficulty in affording food during pregnancy | 1.3 | 0.8 | 0.063 |
| Mother’s opinion of neighbourhood during pregnancy |  |  | <0.001 |
| Very good area | 34.7 | 42.9 |  |
| Fairly good area | 43.6 | 48.5 |  |
| Not very good area | 5.4 | 3.4 |  |
| Bad area | 1.8 | 0.6 |  |
| Separated during pregnancy (partner-reported) | 1.0 | 0.8 | 0.17 |
| Separated during pregnancy (mother-reported) | 0.8 | 0.6 | 0.32 |
| Mother divorced during pregnancy | 3.1 | 1.5 | <0.001 |
| Mother has no one to share feelings with | 0.8 | 0.5 | 0.12 |
| Partner has no one to share feelings with | 0.8 | 0.5 | 0.11 |
| Partner’s hard drug use during pregnancy | 1.1 | 1.2 | 0.5 |
| Partner’s convicted during pregnancy | 1.0 | 0.5 | 0.038 |

ALSPAC, The Avon Longitudinal Study of Parents and Children; SE, standard error; DNAm, DNA methylation; EPDS, Edinburgh Postnatal Depression Scale

^a^ Two-tailed *P* value

### **Table S2.** Adverse childhood experience and Horvath DNA methylation age acceleration

| **ACE** | **Unadjusted mean difference in Horvath DNA methylation age acceleration, years (±95% CI)** | **LR test for sex (χ^2^, *P* value), dof=1** | **Adjusted^a^ mean difference in Horvath DNA methylation age acceleration, years (±95% CI)** | **LR test for sex (χ^2^, *P* value), dof=1** |
| --- | --- | --- | --- | --- |
| Count of ACEs |  |  |  |  |
| None | 0 |  | 0 |  |
| One | -0.23 (-1.00, 0.54) | 3.83, 0.050 | -0.13 (-0.91, 0.65) | 12.88, 0.001 |
| Two or three | -0.02 (-0.74, 0.69) | 3.70, 0.055 | 0.10 (-0.67, 0.87) | 12.59, 0.001 |
| Four or more | 0.58 (-0.28, 1.44) | 3.63, 0.057 | 0.74 (-0.24, 1.71) | 12.70, 0.001 |
| Individual ACE exposure |  |  |  |  |
| Bullying | -0.18 (-0.88, 0.53) | 3.82, 0.051 | -0.11 (-0.84, 0.62) | 12.72, 0.001 |
| Emotional abuse | 0.26 (-0.42, 0.94) | 3.79, 0.051 | 0.31 (-0.42, 1.04) | 12.79, 0.001 |
| Emotional neglect | 0.31 (-0.37, 0.99) | 3.65, 0.056 | 0.13 (-0.57, 0.84) | 12.63, 0.001 |
| Parent mental health problem | 0.15 (-0.39, 0.70) | 3.89, 0.049 | 0.29 (-0.32, 0.90) | 13.31, 0.001 |
| Parent convicted | 0.43 (-0.46, 1.33) | 3.85, 0.049 | 0.37 (-0.59, 1.32) | 13.00, 0.001 |
| Parental separation | 0.54 (-0.11, 1.19) | 3.36, 0.067 | 0.66 (-0.07, 1.40) | 11.96, 0.002 |
| Physical abuse | 0.64 (-0.12, 1.40) | 3.78, 0.052 | 0.67 (-0.13, 1.46) | 12.50, 0.002 |
| Sexual abuse | 0.50 (-1.00, 2.00) | 3.99, 0.047 | 0.50 (-1.06, 2.07) | 13.45, 0.001 |
| Household substance abuse | 0.60 (-0.26, 1.45) | 3.71, 0.055 | -0.11 (-0.84, 0.62) | 12.66, 0.002 |
| Violence in household | 0.23 (-0.46, 0.92) | 3.72, 0.054 | 0.31 (-0.42, 1.04) | 12.71, 0.001 |

ACE, adverse childhood experience; 95% CI, 95% confidence interval; LR test, likelihood ratio test; dof, degree of freedom

^a^ Adjusted for smoking status at time of DNA methylation measurement, maternal BMI, maternal smoking during pregnancy, maternal age at delivery, maternal depression during pregnancy, partner’s depression during pregnancy, mother’s highest education qualification, household’s highest socioeconomic class

### **Table S3.** Adverse childhood experience and baseline plasma cortisol

| **ACE** | **Unadjusted mean difference in cortisol, nmol/L (±95% CI)** | **LR test for sex (χ^2^, *P* value), dof=1** | **Adjusted^a^ mean difference in cortisol, nmol/L (±95% CI)** | **LR test for sex (χ^2^, *P* value), dof=1** |
| --- | --- | --- | --- | --- |
| Count of ACEs |  |  |  |  |
| None | 0 |  | 0 |  |
| One | 13.73 (-34.26, 61.71) | 18.41, 0.003 | 17.44 (-31.60, 66.47) | 20.48, 0.002 |
| Two or three | 25.41 (-21.90, 72.72) | 18.12, 0.003 | 34.22 (-16.06, 84.51) | 19.98, 0.002 |
| Four or more | 13.50 (-48.00, 75.00) | 18.42, 0.003 | 26.38 (-41.48, 94.25) | 20.53, 0.002 |
| Individual ACE exposure |  |  |  |  |
| Bullying | -15.96 (-67.69, 35.76) | 18.17, 0.004 | -8.63 (-63.50, 46.24) | 20.37, 0.002 |
| Emotional abuse | 29.99 (-22.23, 82.21) | 18.30, 0.003 | 39.87 (-17.04, 96.78) | 20.28, 0.003 |
| Emotional neglect | -22.78 (-73.45, 27.88) | 18.15, 0.003 | -22.78 (-75.49, 29.94) | 20.22, 0.002 |
| Parent mental health problem | 14.46 (-24.80, 53.72) | 17.98, 0.004 | 26.24 (-17.66, 70.14) | 19.39, 0.002 |
| Parent convicted | 18.44 (-58.73, 95.61) | 18.27, 0.003 | 28.92 (-51.79, 109.63) | 20.18, 0.002 |
| Parental separation | 8.72 (-37.67, 55.12) | 18.79, 0.003 | 2.59 (-48.02, 53.20) | 20.88, 0.002 |
| Physical abuse | 32.65 (-20.30, 85.59) | 18.44, 0.003 | 37.35 (-17.72, 92.43) | 20.39, 0.002 |
| Sexual abuse | -10.83 (-126.41, 104.75) | 18.68, 0.003 | -8.35 (-123.16, 106.45) | 20.70, 0.002 |
| Household substance abuse | -31.27 (-97.40, 34.86) | 18.37, 0.003 | -36.56 (-106.24, 33.13) | 20.70, 0.002 |
| Violence in household | -6.64 (-56.57, 43.29) | 18.40, 0.003 | -6.59 (-60.37, 47.20) | 20.50, 0.002 |

ACE, adverse childhood experience; 95% CI, 95% confidence interval; LR test, likelihood ratio test; dof, degree of freedom

^a^ Adjusted for body mass index at time of cortisol measurement, maternal body mass index, maternal smoking during pregnancy, maternal age at delivery, maternal depression during pregnancy, partner’s depression during pregnancy, mother’s highest education qualification, household’s highest socioeconomic class

### **Table S4.** Adverse childhood experience and Hannum estimated DNA methylation age acceleration **stratified by sex**

| **ACE** | **Unadjusted mean difference in Hannum DNA methylation age acceleration, years (±95% CI)** | | **Adjusted^a^ mean difference in Hannum DNA methylation age acceleration, years (±95% CI)** | |
| --- | --- | --- | --- | --- |
|  | **Girls** | **Boys** | **Girls** | **Boys** |
| Count of ACEs |  |  |  |  |
| None | 0 | 0 | 0 | 0 |
| One | 0.47 (-0.62, 1.56) | 0.47 (-0.65, 1.60) | 0.26 (-0.89, 1.41) | -0.48 (-1.56, 0.60) |
| Two or three | 0.27 (-0.76, 1.31) | -0.34 (-1.39, 0.72) | -0.13 (-1.28, 1.02) | -0.30 (-1.37, 0.78) |
| Four or more | 0.74 (-0.45, 1.92) | -0.39 (-1.61, 0.83) | 0.26 (-1.15, 1.68) | -0.11 (-1.48, 1.26) |
| Individual ACE exposure |  |  |  |  |
| Bullying | 0.04 (-0.97, 1.05) | -0.12 (-1.10, 0.87) | 0.01 (-1.07, 1.09) | -0.26 (-1.33, 0.82) |
| Emotional abuse | 0.85 (-0.14, 1.84) | -1.16 (-2.13, -0.18)^b^ | 0.67 (-0.43, 1.78) | -1.22 (-2.37, -0.07)^b^ |
| Emotional neglect | -0.05 (-1.05, 0.94) | 0.56 (-0.41, 1.53) | 0.14 (-0.95, 1.23) | 0.42 (-0.66, 1.50) |
| Parent mental health problem | 0.29 (-0.49, 1.06) | -0.11 (-0.90, 0.68) | -0.12 (-1.04, 0.80) | 0.24 (-0.71, 1.19) |
| Parent convicted | 0.06 (-1.19, 1.32) | -0.51 (-1.99, 0.96) | -0.33 (-1.76, 1.10) | -0.41 (-1.97, 1.16) |
| Parental separation | 0.48 (-0.50, 1.46) | -0.75 (-1.69, 0.20) | 0.57 (-0.61, 1.75) | -0.45 (-1.56, 0.67) |
| Physical abuse | 0.25 (-0.78, 1.28) | -0.31 (-1.35, 0.73) | 0.07 (-1.04, 1.18) | -0.33 (-1.54, 0.88) |
| Sexual abuse | 1.48 (-0.43, 3.39) | -0.11 (-3.31, 3.10) | 1.33 (-0.71, 3.38) | -0.29 (-3.77, 3.19) |
| Household substance abuse | 0.52 (-0.70, 1.75) | 0.32 (-0.88, 1.52) | 0.37 (-0.99, 1.74) | 0.37 (-1.01, 1.76) |
| Violence in household | -0.76 (-1.74, 0.23) | 0.13 (-0.88, 1.13) | -1.31 (-2.38, -0.23)^b^ | 0.46 (-0.67, 1.60) |

ACE, adverse childhood experience; 95% CI, 95% confidence interval

^a^ Adjusted for white blood cell composition, smoking status at time of DNA methylation measurement, maternal body mass index, maternal smoking during pregnancy, maternal age at delivery, maternal depression during pregnancy, partner’s depression during pregnancy, mother’s highest education qualification, household’s highest socioeconomic class

^b^ Two-tailed *P* value <0.05

### **Table S5.** Adverse childhood experience and baseline plasma cortisol **stratified by sex**

| **Adverse childhood experience** | **Unadjusted mean difference in cortisol, nmol/L (±95% CI)** | | **Adjusted^a^ mean difference in cortisol, nmol/L (±95% CI)** | |
| --- | --- | --- | --- | --- |
|  | **Girls** | **Boys** | **Girls** | **Boys** |
| Count of ACEs |  |  |  |  |
| None | 0 | 0 | 0 | 0 |
| One | 6.69 (-70.77, 84.14) | 21.93 (-32.08, 75.94) | 39.56 (-35.68, 114.81) | 10.70 (-42.37, 63.76) |
| Two or three | 16.43 (-60.02, 92.89) | 31.89 (-21.72, 85.50) | 33.32 (-43.82, 110.47) | 23.75 (-33.87, 81.37) |
| Four or more | 17.78 (-80.96, 116.53) | 10.74 (-58.51, 79.99) | 41.20 (-62.04, 144.45) | 8.79 (-69.39, 86.96) |
| Individual ACE exposure |  |  |  |  |
| Bullying | -8.46 (-94.46, 77.54) | -17.22 (-73.18, 38.74) | 15.27 (-74.26, 104.79) | -11.66 (-70.56, 47.23) |
| Emotional abuse | 37.61 (-51.64, 126.85) | 19.89 (-38.28, 78.06) | 35.31 (-61.74, 132.36) | 19.09 (-48.40, 86.57) |
| Emotional neglect | -40.65 (-125.42, 44.11) | -1.42 (-59.72, 56.87) | -38.48 (-119.46, 42.51) | 5.69 (-56.01, 67.40) |
| Parent mental health problem | 1.84 (-62.02, 65.70) | 21.44 (-23.79, 66.68) | 30.59 (-37.22, 98.40) | 26.42 (-29.49, 82.33) |
| Parent convicted | 37.08 (-90.90, 165.06) | -7.57 (-90.35, 75.21) | 49.52 (-91.94, 190.98) | -13.00 (-98.82, 72.81) |
| Parental separation | 9.25 (-68.25, 86.75) | 15.87 (-40.38, 72.12) | -13.38 (-90.20, 63.44) | 21.35 (-45.05, 87.75) |
| Physical abuse | 42.74 (-46.23, 131.72) | 21.43 (-40.62, 83.49) | 48.15 (-38.18, 134.48) | 12.65 (-55.90, 81.19) |
| Sexual abuse | -24.40 (-176.07, 127.27) | -19.60 (-173.84, 134.65) | -9.12 (-163.73, 145.49) | -27.46 (-184.45, 129.54) |
| Household substance abuse | -12.62 (-120.08, 94.84) | -49.79 (-122.36, 22.78) | -15.93 (-129.60, 97.74) | -66.81 (-144.08, 10.47) |
| Violence in household | -7.89 (-96.84, 81.05) | -4.39 (-59.83, 51.05) | -1.52 (-97.78, 94.75) | -6.13 (-70.35, 58.09) |

95% CI, 95% confidence interval; ACE, adverse childhood experience

^a^ Adjusted for cortisol sampling time, body mass index at time of cortisol measurement, maternal body mass index, maternal smoking during pregnancy, maternal age at delivery, maternal depression during pregnancy, partner’s depression during pregnancy, mother’s highest education qualification, household’s highest socioeconomic class

### **Table S6.** Distribution of observed (non-imputed) and imputed data (N=974)

| **Variable** | **% data imputed** | **Mean (SE) or % in observed data** | **Mean (SE) or % in imputed data** |
| --- | --- | --- | --- |
| Chronological age | 0 | 17.14 (0.03) | 17.14 (0.03) |
| DNA methylation age acceleration |  |  |  |
| Hannum (years) | 0 | 0.00 (0.13) | 0.00 (0.13) |
| Horvath (years) | 0 | 0.00 (0.13) | 0.00 (0.13) |
| Plasma cortisol (nmol/L) | 56.1 | 485.79 (9.13) | 494.95 (9.97) |
| Count of adverse childhood experiences | NA |  |  |
| None |  | 52.0 | 21.7 |
| One |  | 19.9 | 25.1 |
| Two or three |  | 21.3 | 34.3 |
| Four or more |  | 6.9 | 18.8 |
| Individual adverse childhood experience exposure |  |  |  |
| Bullying | 4.4 | 14.5 | 15.7 |
| Emotional abuse | 19.3 | 14.2 | 22.4 |
| Emotional neglect | 0.5 | 15.6 | 15.7 |
| Parent mental health problem | 21.6 | 36.7 | 50.0 |
| Parent convicted | 20.9 | 6.1 | 12.3 |
| Parental separation | 16.9 | 16.2 | 23.3 |
| Physical abuse | 19.9 | 10.5 | 17.9 |
| Sexual abuse | 5.2 | 2.2 | 3.5 |
| Household substance abuse | 15.0 | 8.1 | 13.6 |
| Violence in household | 21.4 | 13.8 | 21.6 |
| **Covariates** | **% data imputed** | **Mean (SE) or % in observed data** | **Mean (SE) or % in imputed data** |
| White blood cell composition |  |  |  |
| CD8+ naïve cells | 0 | 323.92 (1.77) | 323.92 (1.77) |
| CD4+ naïve cells | 0 | 580.67 (4.21) | 580.67 (4.21) |
| CD8+ T cells | 0 | 0.06 (<0.01) | 0.06 (<0.01) |
| CD4+ T cells | 0 | 0.19 (<0.01) | 0.19 (<0.01) |
| Natural killer cells | 0 | 0.07 (<0.01) | 0.07 (<0.01) |
| B cells | 0 | 0.10 (<0.01) | 0.10 (<0.01) |
| Monocytes | 0 | 0.06 (<0.01) | 0.06 (<0.01) |
| Granulocytes | 0 | 0.51 (<0.01) | 0.51 (<0.01) |
| Cortisol sampling time from 08:00 (minutes) | 56.1 | 43.83 (0.91) | 43.72 (1.22) |
| Body mass index (kg/m^2^) at cortisol sampling | 8.0 | 21.37 (0.11) | 21.42 (0.12) |
| Smoking | 0 |  |  |
| No |  | 74.5 | 74.5 |
| Yes |  | 13.3 | 13.3 |
| NA |  | 12.1 | 12.1 |
| Maternal smoking during pregnancy | 9.0 |  |  |
| No |  | 78.2 | 83.9 |
| Early |  | 2.4 | 3.7 |
| Sustained |  | 10.4 | 12.2 |
| Child’s ethnicity is non-white | 3.8 | 2.7 | 2.7 |
| Child’s birth weight (g) | 2.1 | 3492.49 (15.85) | 3493.36 (15.95) |
| Child’s gestational age at delivery (weeks) | 0.6 | 39.61 (0.05) | 39.61 (0.37) |
| Maternal pre-pregnancy weight (kg) | 6.6 | 61.61 (0.35) | 61.68 (0.37) |
| Maternal body mass index during pregnancy (kg/m^2^) | 7.4 | 22.78 (0.12) | 22.80 (0.13) |
| Mother’s home ownership status during pregnancy | 2.9 |  |  |
| Mortgaged |  | 83.8 | 86.3 |
| Owned |  | 1.7 | 1.8 |
| Council rented |  | 5.7 | 5.9 |
| Rent (private and furnished) |  | 2.3 | 2.3 |
| Rent (private and unfurnished) |  | 1.2 | 1.3 |
| Housing authority rented |  | <0.5 | <0.5 |
| Other |  | 2.1 | 2.1 |
| Maternal age at delivery (years) | 0.6 | 29.55 (0.14) | 29.55 (0.14) |
| Maternal parity | 3.3 | 0.73 (0.27) | 0.77 (0.03) |
| Mother’s marital status during pregnancy | 1.6 |  |  |
| Never married |  | 11.9 | 12.2 |
| Widowed |  | <0.5 | <0.5 |
| Divorced |  | 3.6 | 3.7 |
| Separated |  | 1.1 | 1.2 |
| 1st marriage |  | 76.3 | 77.4 |
| Marriage 2 or 3 |  | 5.3 | 5.4 |
| Mother’s highest education qualification (self-reported) | 2.2 |  |  |
| Certificate of secondary education |  | 8.5 | 9.0 |
| Vocational |  | 7.2 | 7.6 |
| Ordinary level |  | 33.2 | 33.7 |
| Advanced level |  | 28.9 | 29.2 |
| Degree |  | 20.1 | 20.5 |
| Mother became homeless during pregnancy | 5.0 | 1.7 | 3.0 |
| Mother’s depression score (EPDS) at 18 weeks gestation | 6.2 | 6.33 (0.15) | 6.41 (0.16) |
| Mother’s depression score (EPDS) at 32 weeks gestation | 4.1 | 6.56 (0.15) | 6.69 (0.16) |
| Partner’s depression score (EPDS) at 18 weeks gestation | 18.4 | 4.12 (0.14) | 4.64 (0.34) |
| Household social class at 18 weeks gestation | 3.7 |  |  |
| I - Professional |  | 14.6 | 15.1 |
| II - Managerial and technical |  | 41.4 | 42.9 |
| IIINM - Skilled non-manual |  | 26.5 | 27.6 |
| IIIM - Skilled manual |  | 8.5 | 8.8 |
| IV - Partly skilled |  | 4.8 | 5.0 |
| V - Unskilled |  | 0.5 | 0.5 |
| **Auxiliary imputation variables** | **% data imputed** | **Mean (SE) or % in observed data** | **Mean (SE) or % in imputed data** |
| Poor parent-child bonding | 16.7 | 14.3 | 20.9 |
| Financial difficulties | 13.3 | 9.3 | 14.6 |
| Intimate partner violence | 41.0 | 5.3 | 15.6 |
| Neighbourhood | 4.9 | 2.7 | 4.2 |
| Child physical illness | 60.5 | 4.7 | 25.3 |
| Parent physical illness | 83.0 | 8.3 | 51.1 |
| Low socioeconomic status | 27.1 | 7.1 | 15.3 |
| Child lacking social support | 4.1 | 7.3 | 8.5 |
| Parent lacking social support | 11.0 | 8.5 | 11.7 |
| Poor parent-child bonding | 16.7 | 14.3 | 20.9 |
| Partner’s highest education qualification (mother-reported) | 3.5 |  |  |
| Certificate of secondary education |  | 14.8 | 16.1 |
| Vocational |  | 7.2 | 7.8 |
| Ordinary level |  | 21.1 | 21.7 |
| Advanced level |  | 28.0 | 28.6 |
| Degree |  | 25.4 | 25.9 |
| Partner became homeless during pregnancy | 18.4 | 0.5 | 4.9 |
| Difficulty in affording heating during pregnancy | 3.7 | 2.0 | 3.4 |
| Difficulty in affording food during pregnancy | 3.7 | 0.8 | 2.0 |
| Mother’s opinion of neighbourhood during pregnancy | 4.6 |  |  |
| Very good area |  | 42.9 | 44.3 |
| Fairly good area |  | 48.5 | 49.9 |
| Not very good area |  | 3.4 | 4.4 |
| Bad area |  | 0.6 | 1.4 |
| Separated during pregnancy (partner-reported) | 18.4 | 0.8 | 6.3 |
| Separated during pregnancy (mother-reported) | 6.6 | 1.5 | 3.5 |
| Mother divorced during pregnancy | 6.7 | 0.6 | 2.1 |
| Mother has no one to share feelings with | 3.0 | 0.5 | 1.3 |
| Partner has no one to share feelings with | 19.2 | 0.5 | 6.2 |
| Partner’s hard drug use during pregnancy | 19.6 | 1.2 | 6.2 |
| Partner’s convicted during pregnancy | 17.6 | 0.5 | 4.9 |

NA, not applicable; SE, standard error; EPDS, Edinburgh Postnatal Depression Scale

### **Table S7.** Distribution of observed (non-imputed) and imputed data in **girls (N=500)**

| **Variable** | **% data imputed** | **Mean (SE) or % in observed data** | **Mean (SE) or % in imputed data** |
| --- | --- | --- | --- |
| Chronological age | 0 | 17.11 (0.05) | 17.11 (0.05) |
| DNA methylation age acceleration |  |  |  |
| Hannum (years) | 0 | -0.46 (0.18) | -0.46 (0.18) |
| Horvath (years) | 0 | -0.24 (0.18) | -0.24 (0.18) |
| Plasma cortisol (nmol/L) | 55.8 | 514.46 (14.55) | 522.54 (15.99) |
| Count of adverse childhood experiences | NA |  |  |
| None |  | 51.6 | 21.5 |
| One |  | 19.4 | 24.4 |
| Two or three |  | 22.6 | 35.9 |
| Four or more |  | 6.4 | 18.2 |
| Individual adverse childhood experience exposures |  |  |  |
| Bullying | 3.2 | 13.4 | 14.4 |
| Emotional abuse | 19.2 | 14.0 | 22.9 |
| Emotional neglect | 0.2 | 14.6 | 14.6 |
| Parent mental health problem | 22.0 | 39.2 | 52.7 |
| Parent convicted | 21.8 | 6.4 | 13.1 |
| Parental separation | 18.2 | 13.6 | 20.9 |
| Physical abuse | 19.0 | 10.8 | 18.0 |
| Sexual abuse | 5.8 | 3.2 | 4.9 |
| Household substance abuse | 16.2 | 7.6 | 13.4 |
| Violence in household | 21.8 | 13.8 | 21.3 |
| **Covariates** | **% data imputed** | **Mean (SE) or % in observed data** | **Mean (SE) or % in imputed data** |
| White blood cell composition |  |  |  |
| CD8+ naïve cells | 0 | 321.35 (2.54) | 321.35 (2.54) |
| CD4+ naïve cells | 0 | 587.81 (5.94) | 587.81 (5.94) |
| CD8+ T cells | 0 | 0.06 (<0.01) | 0.06 (<0.01) |
| CD4+ T cells | 0 | 0.20 (<0.01) | 0.20 (<0.01) |
| Natural killer cells | 0 | 0.07 (<0.01) | 0.07 (<0.01) |
| B cells | 0 | 0.09 (<0.01) | 0.09 (<0.01) |
| Monocytes | 0 | 0.06 (<0.01) | 0.06 (<0.01) |
| Granulocytes | 0 | 0.52 (<0.01) | 0.52 (<0.01) |
| Cortisol sampling time from 08:00 (minutes) | 55.8 | 43.48 (1.17) | 44.21 (0.94) |
| Body mass index (kg/m^2^) at cortisol sampling | 8.6 | 21.68 (0.16) | 21.77 (0.16) |
| Smoking | 0 |  |  |
| No |  | 75.6 | 75.6 |
| Yes |  | 12.6 | 12.6 |
| NA |  | 11.8 | 11.8 |
| Maternal smoking during pregnancy | 9.0 |  |  |
| No |  | 78.0 | 83.1 |
| Early |  | 2.6 | 4.2 |
| Sustained |  | 10.4 | 12.6 |
| Child’s ethnicity is non-white | 4.6 | 2.2 | 2.2 |
| Child’s birth weight (g) | 2.6 | 3418.30 (20.95) | 3421.47 (20.85) |
| Child’s gestational age at delivery (weeks) | 1.0 | 39.68 (0.07) | 39.68 (0.07) |
| Maternal pre-pregnancy weight (kg) | 6.8 | 61.37 (0.45) | 61.28 (0.45) |
| Maternal body mass index during pregnancy (kg/m^2^) | 7.6 | 22.69 (0.16) | 22.69 (0.16) |
| Mother’s home ownership status during pregnancy | 4.0 |  |  |
| Mortgaged |  | 83.2 | 86.7 |
| Owned |  | 1.0 | 1.1 |
| Council rented |  | 6.6 | 6.9 |
| Rent (private and furnished) |  | 2.0 | 2.1 |
| Rent (private and unfurnished) |  | 1.2 | 1.2 |
| Housing authority rented |  | <1.0 | <1.0 |
| Other |  | 1.8 | 1.9 |
| Maternal age at delivery (years) | 1.0 | 29.24 (0.19) | 29.26 (0.20) |
| Maternal parity | 4.4 | 0.71 (0.04) | 0.77 (0.04) |
| Mother’s marital status during pregnancy | 2.2 |  |  |
| Never married |  | 13.0 | 13.4 |
| Widowed |  | <1.0^a^ | <1.0^a^ |
| Divorced |  | 2.8 | 3.0 |
| Separated |  | 1.0 | 1.2 |
| 1st marriage |  | 77.4 | 78.8 |
| Marriage 2 or 3 |  | 3.6 | 3.7 |
| Mother’s highest education qualification (self-reported) | 2.8 |  |  |
| Certificate of secondary education |  | 9.4 | 9.9 |
| Vocational |  | 6.2 | 6.7 |
| Ordinary level |  | 33.6 | 34.5 |
| Advanced level |  | 28.4 | 28.8 |
| Degree |  | 19.6 | 20.1 |
| Mother became homeless during pregnancy | 4.6 | 1.4 | 2.6 |
| Mother’s depression score (EPDS) at 18 weeks gestation | 6.2 | 6.11 (0.21) | 6.11 (0.21) |
| Mother’s depression score (EPDS) at 32 weeks gestation | 4.4 | 6.34 (0.21) | 6.47 (0.21) |
| Partner’s depression score (EPDS) at 18 weeks gestation | 18.6 | 4.05 (0.19) | 4.55 (0.19) |
| Household social class at 18 weeks gestation | 4.0 |  |  |
| I - Professional |  | 12.6 | 13.1 |
| II - Managerial and technical |  | 41.8 | 43.5 |
| IIINM - Skilled non-manual |  | 28.6 | 29.9 |
| IIIM - Skilled manual |  | 8.2 | 8.5 |
| IV - Partly skilled |  | 4.4 | 4.6 |
| V - Unskilled |  | <1.0 | <1.0 |
| **Auxiliary imputation variables** | **% data imputed** | **Mean (SE) or % in observed data** | **Mean (SE) or % in imputed data** |
| Poor parent-child bonding | 18.4 | 12.4 | 19.7 |
| Financial difficulties | 14.6 | 9.8 | 15.2 |
| Intimate partner violence | 39.0 | 4.6 | 14.9 |
| Neighbourhood | 6.0 | 2.6 | 4.4 |
| Child physical illness | 60.0 | 3.8 | 23.1 |
| Parent physical illness | 83.0 | 9.2 | 53.8 |
| Low socioeconomic status | 27.2 | 7.6 | 16.1 |
| Child lacking social support | 3.6 | 6.6 | 7.6 |
| Parent lacking social support | 11.4 | 9.2 | 12.2 |
| Partner’s highest education qualification (mother-reported) | 4.2 |  |  |
| Certificate of secondary education |  | 16.4 | 18.0 |
| Vocational |  | 6.8 | 7.7 |
| Ordinary level |  | 21.0 | 21.5 |
| Advanced level |  | 27.6 | 28.2 |
| Degree |  | 24.0 | 24.6 |
| Partner became homeless during pregnancy | 18.2 | <1.0 | 4.5 |
| Difficulty in affording heating during pregnancy | 3.6 | 2.4 | 3.9 |
| Difficulty in affording food during pregnancy | 3.6 | 1.2 | 2.5 |
| Mother’s opinion of neighbourhood during pregnancy | 6.2 |  |  |
| Very good area |  | 40.0 | 41.9 |
| Fairly good area |  | 49.8 | 51.7 |
| Not very good area |  | 3.6 | 5.0 |
| Bad area |  | 0.4 | 1.4 |
| Separated during pregnancy (partner-reported) | 18.2 | <1.0 | 5.4 |
| Separated during pregnancy (mother-reported) | 7.2 | 1.2 | 3.6 |
| Mother divorced during pregnancy | 7.0 | 0.8 | 2.5 |
| Mother has no one to share feelings with | 3.6 | 0.6 | 1.5 |
| Partner has no one to share feelings with | 19.8 | 0.6 | 6.2 |
| Partner’s hard drug use during pregnancy | 19.8 | 1.2 | 5.8 |
| Partner’s convicted during pregnancy | 17.6 | 0.8 | 4.8 |

SE, standard error; NA, not applicable; EPDS, Edinburgh Postnatal Depression Scale

^a^ This may include zero

### **Table S8.** Distribution of observed (non-imputed) and imputed data in **boys (N=474)**

| **Variable** | **% data imputed** | **Mean (SE) or % in observed data** | **Mean (SE) or % in imputed data** |
| --- | --- | --- | --- |
| Chronological age | 0 | 17.16 (0.05) | 17.16 (0.05) |
| DNA methylation age acceleration |  |  |  |
| Hannum (years) | 0 | 0.48 (0.18) | 0.48 (0.18) |
| Horvath (years) | 0 | 0.25 (0.17) | 0.25 (0.17) |
| Plasma cortisol (nmol/L) | 56.3 | 455.17 (10.35) | 465.85 (11.24) |
| Count of adverse childhood experiences | NA |  |  |
| None |  | 52.3 | 22.0 |
| One |  | 20.5 | 25.9 |
| Two or three |  | 19.8 | 32.7 |
| Four or more |  | 7.4 | 19.4 |
| Individual adverse childhood experience exposures |  |  |  |
| Bullying | 5.7 | 15.6 | 17.1 |
| Emotional abuse | 19.4 | 14.3 | 21.9 |
| Emotional neglect | 0.8 | 16.7 | 16.9 |
| Parent mental health problem | 21.1 | 34.0 | 47.2 |
| Parent convicted | 20.0 | 5.7 | 11.6 |
| Parental separation | 15.6 | 19.0 | 25.9 |
| Physical abuse | 20.9 | 10.1 | 17.8 |
| Sexual abuse | 4.6 | 1.1 | 2.0 |
| Household substance abuse | 13.7 | 8.6 | 13.9 |
| Violence in household | 20.9 | 13.7 | 21.9 |
| **Covariates** | **% data imputed** | **Mean (SE) or % in observed data** | **Mean (SE) or % in imputed data** |
| White blood cell composition |  |  |  |
| CD8+ naïve cells | 0 | 326.62 (2.45) | 326.62 (2.45) |
| CD4+ naïve cells | 0 | 573.12 (5.95) | 573.12 (5.95) |
| CD8+ T cells | 0 | 0.06 (<0.01) | 0.06 (<0.01) |
| CD4+ T cells | 0 | 0.19 (<0.01) | 0.19 (<0.01) |
| Natural killer cells | 0 | 0.08 (<0.01) | 0.08 (<0.01) |
| B cells | 0 | 0.11 (<0.01) | 0.11 (<0.01) |
| Monocytes | 0 | 0.07 (<0.01) | 0.07 (<0.01) |
| Granulocytes | 0 | 0.50 (<0.01) | 0.50 (<0.01) |
| Cortisol sampling time from 08:00 (minutes) | 56.3 | 44.20 (1.40) | 43.58 (1.20) |
| Body mass index (kg/m^2^) | 7.4 | 21.05 (0.15) | 21.11 (0.15) |
| Smoking | 0 |  |  |
| NA |  | 73.4 | 73.4 |
| No |  | 14.1 | 14.1 |
| Yes |  | 12.4 | 12.4 |
| Maternal smoking during pregnancy | 9.1 |  |  |
| No |  | 78.5 | 84.9 |
| Early |  | 2.1 | 3.2 |
| Sustained |  | 10.3 | 11.9 |
| Child’s ethnicity is non-white | 3.0 | 3.2 | 3.2 |
| Child’s birth weight (g) | 1.5 | 3569.86 (23.39) | 3570.72 (23.42) |
| Child’s gestational age at delivery (weeks) | 0.2 | 39.54 (0.07) | 39.53 (0.07) |
| Maternal pre-pregnancy weight (kg) | 6.3 | 61.87 (0.54) | 62.31 (0.54) |
| Maternal body mass index during pregnancy (kg/m^2^) | 7.2 | 22.88 (0.18) | 22.99 (0.18) |
| Mother’s home ownership status during pregnancy | 1.7 |  |  |
| Mortgaged |  | 84.4 | 85.8 |
| Owned |  | 2.5 | 2.6 |
| Council rented |  | 4.9 | 4.9 |
| Rent (private and furnished) |  | 2.5 | 2.6 |
| Rent (private and unfurnished) |  | 1.3 | 1.3 |
| Housing authority rented |  | <1.0 | <1.0 |
| Other |  | 2.3 | 2.4 |
| Maternal age at delivery (years) | 0.2 | 29.87 (0.21) | 29.86 (0.21) |
| Maternal parity | 2.1 | 0.77 (0.04) | 0.78 (0.04) |
| Mother’s marital status during pregnancy | 1.1 |  |  |
| Never married |  | 10.8 | 10.9 |
| Widowed |  | <1.0 | <1.0 |
| Divorced |  | 4.4 | 4.5 |
| Separated |  | 1.3 | 1.3 |
| 1st marriage |  | 75.1 | 75.9 |
| Marriage 2 or 3 |  | 7.2 | 7.2 |
| Mother’s highest education qualification (self-reported) | 1.5 |  |  |
| Certificate of secondary education |  | 7.6 | 8.1 |
| Vocational |  | 8.2 | 8.5 |
| Ordinary level |  | 32.7 | 32.9 |
| Advanced level |  | 29.3 | 30.0 |
| Degree |  | 20.7 | 20.9 |
| Mother became homeless during pregnancy | 5.5 | 2.1 | 3.5 |
| Mother’s depression score (EPDS) at 18 weeks gestation | 6.1 | 6.56 (0.22) | 6.59 (0.25) |
| Mother’s depression score (EPDS) at 32 weeks gestation | 3.8 | 6.79 (0.22) | 6.87 (0.24) |
| Partner’s depression score (EPDS) at 18 weeks gestation | 18.1 | 4.19 (0.20) | 4.89 (0.63) |
| Household social class at 18 weeks gestation | 3.4 |  |  |
| I - Professional |  | 16.7 | 17.3 |
| II - Managerial and technical |  | 40.9 | 42.3 |
| IIINM - Skilled non-manual |  | 24.3 | 25.1 |
| IIIM - Skilled manual |  | 8.9 | 9.2 |
| IV - Partly skilled |  | 5.3 | 5.4 |
| V - Unskilled |  | <1.0 | <1.0 |
| **Auxiliary imputation variables** | **% data imputed** | **Mean (SE) or % in observed data** | **Mean (SE) or % in imputed data** |
| Poor parent-child bonding | 15.0 | 16.2 | 22.2 |
| Financial difficulties | 12.0 | 8.9 | 13.9 |
| Intimate partner violence | 43.0 | 6.1 | 16.3 |
| Neighbourhood | 3.8 | 2.7 | 4.0 |
| Child physical illness | 61.0 | 5.7 | 27.6 |
| Parent physical illness | 82.9 | 7.4 | 48.3 |
| Low socioeconomic status | 27.0 | 6.5 | 14.6 |
| Child lacking social support | 4.6 | 8.0 | 9.3 |
| Parent lacking social support | 10.5 | 7.8 | 11.2 |
| Partner’s highest education qualification (mother-reported) | 2.7 |  |  |
| Certificate of secondary education |  | 13.1 | 14.0 |
| Vocational |  | 7.6 | 8.0 |
| Ordinary level |  | 21.3 | 21.9 |
| Advanced level |  | 28.5 | 28.9 |
| Degree |  | 26.8 | 27.3 |
| Partner became homeless during pregnancy | 18.6 | <1.0 | 5.3 |
| Difficulty in affording heating during pregnancy | 3.8 | 1.5 | 2.8 |
| Difficulty in affording food during pregnancy | 3.8 | <1.0 | 1.4 |
| Mother’s opinion of neighbourhood during pregnancy | 3.0 |  |  |
| Very good area |  | 46.0 | 46.9 |
| Fairly good area |  | 47.0 | 48.0 |
| Not very good area |  | 3.2 | 3.7 |
| Bad area |  | <1.0 | 1.3 |
| Separated during pregnancy (partner-reported) | 18.6 | <1.0 | 7.3 |
| Separated during pregnancy (mother-reported) | 6.1 | <1.0 | 3.5 |
| Mother divorced during pregnancy | 6.1 | 1.9 | 1.7 |
| Mother has no one to share feelings with | 2.3 | <1.0 | 1.1 |
| Partner has no one to share feelings with | 18.6 | <1.0 | 6.1 |
| Partner’s hard drug use during pregnancy | 19.4 | 1.3 | 6.7 |
| Partner’s convicted during pregnancy | 17.5 | <1.0 | 5.1 |

SE, standard error; NA, not applicable; EPDS, Edinburgh Postnatal Depression Scale

### **Table S9.** Phrasing and criteria for adversity questions in the Avon Longitudinal Study of Parents and Children

| **Adverse childhood experience** | **Phrasing** | **Criteria** |
| --- | --- | --- |
| Bullying | Overt bullying victim items including: child had personal belongings stolen, threatened, blackmailed, hit, beaten up | Weekly |
|  | Relational bullying victim items including: friends tried to get teenager to do things they didn't want to or told lies about child | Weekly |
|  | Child has been bullied | All the time |
|  | Child upset by name calling, exclusion from groups or bullying | Most days |
|  | Someone threatened or blackmailed teenager | Weekly |
|  | Friends tried to get teenager to do things they didn't want to or told lies about teenager | Weekly |
| Emotional abuse | Mother or partner was emotionally cruel to child | Yes |
|  | Adult in family shouted or said hurtful or insulting things to child before age of 11 | Very often |
| Emotional neglect | Carer knows who friends are | Never |
|  | Carer asks or starts conversation about free time or what happened at school | Never |
|  | Carer takes time to listen when teenager talks about what happened in free time | Never |
|  | Child can discuss problems with anyone in their family | Very difficult |
|  | Parent or carer talked about child's experiences at school, with friends or things that are troubling | Never |
|  | Child feels left out of things | Always |
|  | Child feels understood by parents | Not |
|  | When growing up there was someone to take child to the doctor if needed | Never |
|  | Someone in family made child feel important or special before age of 11 | Never |
|  | Carer knows what child does with other children | Nothing |
| Parent mental health problem | Has hurt themselves on purpose | Yes |
|  | Has attempted suicide | Yes |
|  | Has taken medication for anxiety or depression | Yes |
|  | Edinburgh Postnatal Depression Scale | >12 |
|  | Schizophrenia | Yes, either current or ever |
|  | Bulimia, anorexia nervosa | Yes, recently |
|  | Ever admitted to hospital for psychiatric or mental health problems | Yes |
| Parent convicted | Court conviction | Yes |
|  | Convicted of an offence | Yes |
| Parental separation | Parent reports divorce/separation | Yes |
|  | Child reports parents have divorced/separated | Yes |
|  | Child reports parent still has the same partner/husband | No |
| Physical abuse | Mother or partner was physically cruel to child | Yes |
|  | Adult in family pushed, grabbed, shoved/smacked to discipline respondent before age of 11 | Often |
|  | When growing up people in child's family hit them so hard that it left them with bruises or marks | Yes |
|  | Adult in family kicked, punched, hit respondent (so hard it left bruises or marks) before age of 11 | Yes |
| Sexual abuse | Sexually abused | Yes |
|  | When growing up someone molested child (sexually) | Yes |
|  | Touched in a sexual way by adult or older child, or was forced to touch adult or older child in a sexual way before age of 11 | Yes |
|  | Adult or older child forced, or attempted to force, respondent into any sexual activity by threatening or holding respondent down or hurting respondent in some way before age of 11 | Yes |
| Household substance abuse | Smoked cannabis | Every day |
|  | Hard drug use (including crack, heroin, amphetamine, opiate, cocaine, methadone, meth) | Yes |
|  | Hard drug addiction | Yes, recently |
|  | Alcoholism or drink problem | Yes, ever; yes, saw doctor |
|  | Alcohol Use Disorders Identification Test score | >8 |
| Violence in household (parents) | Physically cruel | Yes, affected them |
|  | Kicked, bitten or hit each other | Yes |
|  | Physically twisted arm | Yes |
|  | Throw(n) bodily | Yes |
|  | Beaten each other up | Yes |
|  | Choke or strangle each other | Yes |
|  | Threatened each other with knife | Yes |
|  | Used knife or other weapon on each other | Yes |

### **Table S10.** Distribution of covariates and auxiliary imputation variables

| **Covariates** | **Mean (SE) or % in girls** | **Mean (SE) or % in boys** | ***P* value^a^** |
| --- | --- | --- | --- |
| White blood cell composition |  |  |  |
| CD8+ naïve cells | 321.35 (2.54) | 326.62 (2.45) | 0.14 |
| CD4+ naïve cells | 587.81 (5.94) | 573.12 (5.95) | 0.08 |
| CD8+ T cells | 0.06 (<0.01) | 0.06 (<0.01) | 0.37 |
| CD4+ T cells | 0.20 (<0.01) | 0.19 (<0.01) | 0.05 |
| Natural killer cells | 0.07 (<0.01) | 0.08 (<0.01) | <0.01 |
| B cells | 0.09 (<0.01) | 0.11 (<0.01) | <0.01 |
| Monocytes | 0.06 (<0.01) | 0.07 (<0.01) | <0.01 |
| Granulocytes | 0.52 (<0.01) | 0.50 (<0.01) | <0.01 |
| Cortisol sampling time from 08:00 (minutes) | 44.21 (0.94) | 43.58 (1.20) | 0.68 |
| Body mass index (kg/m^2^) at cortisol sampling | 21.77 (0.16) | 21.11 (0.15) | <0.01 |
| Smoking status at DNA methylation measurement |  |  | 0.46 |
| No | 75.6 | 73.4 |  |
| Yes | 12.6 | 14.1 |  |
| NA | 11.8 | 12.4 |  |
| Maternal smoking during pregnancy |  |  | 0.54 |
| No | 83.1 | 84.9 |  |
| Early | 4.2 | 3.2 |  |
| Sustained | 12.6 | 11.9 |  |
| Child’s ethnicity is non-white | 2.2 | 3.2 | 0.67 |
| Child’s birth weight (g) | 3421.47 (20.85) | 3570.72 (23.42) | <0.01 |
| Child’s gestational age at delivery (weeks) | 39.68 (0.07) | 39.53 (0.07) | 0.12 |
| Maternal pre-pregnancy weight (kg) | 61.28 (0.45) | 62.31 (0.54) | 0.14 |
| Maternal body mass index during pregnancy (kg/m^2^) | 22.69 (0.16) | 22.99 (0.18) | 0.21 |
| Mother’s home ownership status during pregnancy |  |  | 0.70 |
| Mortgaged | 86.7 | 85.8 |  |
| Owned | 1.1 | 2.6 |  |
| Council rented | 6.9 | 4.9 |  |
| Rent (private and furnished) | 2.1 | 2.6 |  |
| Rent (private and unfurnished) | 1.2 | 1.3 |  |
| Housing authority rented | <1.0 | <1.0 |  |
| Other | 1.9 | 2.4 |  |
| Maternal age at delivery (years) | 29.26 (0.20) | 29.86 (0.21) | 0.04 |
| Maternal parity | 0.77 (0.04) | 0.78 (0.04) | 0.89 |
| Mother’s marital status during pregnancy |  |  | 0.16 |
| Never married | 13.4 | 10.9 |  |
| Widowed | <1.0^b^ | <1.0 |  |
| Divorced | 3.0 | 4.5 |  |
| Separated | 1.2 | 1.3 |  |
| 1st marriage | 78.8 | 75.9 |  |
| Marriage 2 or 3 | 3.7 | 7.2 |  |
| Mother’s highest education qualification |  |  | 0.62 |
| Certificate of secondary education | 9.9 | 8.1 |  |
| Vocational | 6.7 | 8.5 |  |
| Ordinary level | 34.5 | 32.9 |  |
| Advanced level | 28.8 | 30.0 |  |
| Degree | 20.1 | 20.9 |  |
| Mother became homeless during pregnancy | 2.6 | 3.5 | 0.51 |
| Mother’s depression score (EPDS) at 18 weeks gestation | 6.11 (0.21) | 6.59 (0.25) | 0.20 |
| Mother’s depression score (EPDS) at 32 weeks gestation | 6.47 (0.21) | 6.87 (0.24) | 0.10 |
| Partner’s depression score (EPDS) 18 weeks gestation | 4.55 (0.19) | 4.89 (0.63) | 0.03 |
| Household social class at 18 weeks gestation |  |  | 0.31 |
| I - Professional | 13.1 | 17.3 |  |
| II - Managerial and technical | 43.5 | 42.3 |  |
| IIINM - Skilled non-manual | 29.9 | 25.1 |  |
| IIIM - Skilled manual | 8.5 | 9.2 |  |
| IV - Partly skilled | 4.6 | 5.4 |  |
| V - Unskilled | <1.0 | <1.0 |  |
| **Auxiliary imputation variables** | **Mean (SE) or % in girls** | **Mean (SE) or % in boys** | ***P* value^a^** |
| Poor parent-child bonding | 19.7 | 22.2 | 0.41 |
| Financial difficulties | 15.2 | 13.9 | 0.64 |
| Intimate partner violence | 14.9 | 16.3 | 0.67 |
| Neighbourhood | 4.4 | 4.0 | 0.79 |
| Child physical illness | 23.1 | 27.6 | 0.32 |
| Parent physical illness | 53.8 | 48.3 | 0.41 |
| Low socioeconomic status | 16.1 | 14.6 | 0.62 |
| Child lacking social support | 7.6 | 9.3 | 0.38 |
| Parent lacking social support | 12.2 | 11.2 | 0.64 |
| Partner’s highest education qualification (mother-reported) |  |  | 0.93 |
| Certificate of secondary education | 18.0 | 14.0 |  |
| Vocational | 7.7 | 8.0 |  |
| Ordinary level | 21.5 | 21.9 |  |
| Advanced level | 28.2 | 28.9 |  |
| Degree | 24.6 | 27.3 |  |
| Partner became homeless during pregnancy | 4.5 | 5.3 | 0.85 |
| Difficulty in affording heating during pregnancy | 3.9 | 2.8 | 0.41 |
| Difficulty in affording food during pregnancy | 2.5 | 1.4 | 0.36 |
| Mother’s opinion of neighbourhood during pregnancy |  |  | 0.11 |
| Very good area | 41.9 | 46.9 |  |
| Fairly good area | 51.7 | 48.0 |  |
| Not very good area | 5.0 | 3.7 |  |
| Bad area | 1.4 | 1.3 |  |
| Separated during pregnancy (partner-reported) | 5.4 | 7.3 | 0.67 |
| Separated during pregnancy (mother-reported) | 3.6 | 3.5 | 0.96 |
| Mother divorced during pregnancy | 2.5 | 1.7 | 0.59 |
| Mother has no one to share feelings with | 1.5 | 1.1 | 0.67 |
| Partner has no one to share feelings with | 6.2 | 6.1 | 0.90 |
| Partner’s hard drug use during pregnancy | 5.8 | 6.7 | 0.80 |
| Partner convicted during pregnancy | 4.8 | 5.1 | 0.98 |

SE, standard error; EPDS, Edinburgh Postnatal Depression Score

Values are reported as mean (SE) for continuous variables and % for categorical variables.

^a^ Two-tailed *P* value

^b^ This may include zero

### **Table S11.** Variables used in multiple imputation model

| **Variable** | **Type of variable** | **Regression model to predict missing in this variable** | **How variable was entered when used to predict missing in other variables** |
| --- | --- | --- | --- |
| Count of adverse childhood experiences | Categorical (4) | Passive imputation (count of classic adverse childhood experiences) | N/A |
| Bullying | Dichotomous | Logistic regression | Dichotomous |
| Emotional abuse | Dichotomous | Logistic regression | Dichotomous |
| Emotional neglect | Dichotomous | Logistic regression | Dichotomous |
| Parent mental health problem | Dichotomous | Logistic regression | Dichotomous |
| Parent convicted | Dichotomous | Logistic regression | Dichotomous |
| Parental separation | Dichotomous | Logistic regression | Dichotomous |
| Physical abuse | Dichotomous | Logistic regression | Dichotomous |
| Sexual abuse | Dichotomous | Logistic regression | Dichotomous |
| Household substance abuse | Dichotomous | Logistic regression | Dichotomous |
| Violence in household | Dichotomous | Logistic regression | Dichotomous |
| CD8+ naïve cells | Continuous | Linear regression | Continuous |
| CD4+ naïve cells | Continuous | Linear regression | Continuous |
| CD8+ T cells | Continuous | Linear regression | Continuous |
| CD4+ T cells | Continuous | Linear regression | Continuous |
| Natural killer cells | Continuous | Linear regression | Continuous |
| B cells | Continuous | Linear regression | Continuous |
| Monocytes | Continuous | Linear regression | Continuous |
| Granulocytes | Continuous | Linear regression | Continuous |
| Cortisol sampling time | Continuous | Linear regression | Continuous |
| Child’s body mass index at time of cortisol sampling | Continuous | Linear regression | Continuous |
| Child’s smoking at time of DNA methylation sampling | Categorical (3) | Logistic regression | 2 indicator variables |
| Maternal smoking during pregnancy | Categorical (3) | Logistic regression | 2 indicator variables |
| Child’s ethnicity | Categorical (2) | Logistic regression | 1 indicator variable |
| Child’ s birthweight (g) | Continuous | Linear regression | Continuous |
| Child’s gestational age at delivery (weeks) | Continuous | Linear regression | Continuous |
| Maternal pre-pregnancy weight (kg) | Continuous | Linear regression | Continuous |
| Maternal body mass index during pregnancy (kg/m^2^) | Continuous | Linear regression | Continuous |
| Mother’s home ownership during pregnancy | Categorical (7) | Polytomous (unordered) regression | 6 indicator variables |
| Maternal age at delivery (years) | Continuous | Linear regression | Continuous |
| Parity | Continuous | Linear regression | Continuous |
| Mother’s marital status during pregnancy | Categorical (6) | Polytomous (unordered) regression | 5 indicator variables |
| Mother’s self-reported highest educational level | Categorical (5) | Polytomous (unordered) regression | 4 indicator variables |
| Mother became homeless during pregnancy | Dichotomous | Logistic regression | Dichotomous |
| Mother's depression score (EPDS) at 18 weeks gestation | Continuous | Linear regression | Continuous |
| Mother's depression score (EPDS) at 32 weeks gestation | Continuous | Linear regression | Continuous |
| Partner's depression score (EPDS) at 18 weeks | Continuous | Linear regression | Continuous |
| Household social class at 18 weeks gestation | Categorical (6) | Polytomous (unordered) regression | 5 indicator variables |
| Poor parent-child bonding | Dichotomous | Logistic regression | Dichotomous |
| Financial difficulties | Dichotomous | Logistic regression | Dichotomous |
| Intimate partner violence | Dichotomous | Logistic regression | Dichotomous |
| Neighbourhood | Dichotomous | Logistic regression | Dichotomous |
| Child physical illness | Dichotomous | Logistic regression | Dichotomous |
| Parent physical illness | Dichotomous | Logistic regression | Dichotomous |
| Low socioeconomic status | Dichotomous | Logistic regression | Dichotomous |
| Child lacking social support | Dichotomous | Logistic regression | Dichotomous |
| Parent lacking social support | Dichotomous | Logistic regression | Dichotomous |
| Partner’s highest educational level (mother-reported) | Categorical (5) | Polytomous (unordered) regression | 4 indicator variables |
| Partner became homeless during pregnancy | Dichotomous | Logistic regression | Dichotomous |
| Difficulty affording food during pregnancy | Dichotomous | Logistic regression | Dichotomous |
| Difficulty affording heating during pregnancy | Dichotomous | Logistic regression | Dichotomous |
| Mother’s opinion of neighbourhood during pregnancy | Categorical (4) | Polytomous (unordered) regression | 3 indicator variables |
| Partner reported separation since pregnancy | Dichotomous | Logistic regression | Dichotomous |
| Mother reported separation since pregnant | Dichotomous | Logistic regression | Dichotomous |
| Mother divorced since pregnancy | Dichotomous | Logistic regression | Dichotomous |
| Mother has no one to share feelings with | Dichotomous | Logistic regression | Dichotomous |
| Partner has no one to share feelings with | Dichotomous | Logistic regression | Dichotomous |
| Partner hard drug use during pregnancy | Dichotomous | Logistic regression | Dichotomous |
| Partner convicted of an offence during pregnancy | Dichotomous | Logistic regression | Dichotomous |

EPDS, Edinburgh Postnatal Depression Scale

**Table S12.** Adverse childhood experience and Horvath DNA methylation age acceleration with and without adjustment for body mass index

| **Exposure** | **Model 1^a^ mean difference in DNA methylation age acceleration,** **years (±95% CI)** | | **Model 2^b^ mean difference in DNA methylation age acceleration,** **years (±95% CI)** | |
| --- | --- | --- | --- | --- |
|  | **Girls** | **Boys** | **Girls** | **Boys** |
| Count of ACEs |  |  |  |  |
| None | 0 | 0 | 0 | 0 |
| One | 0.32 (-0.81, 1.45) | -0.48 (-1.56, 0.60) | 0.28 (-0.85, 1.42) | -0.48 (-1.57, 0.60) |
| Two or three | 0.68 (-0.42, 1.78) | -0.30 (-1.37, 0.78) | 0.66 (-0.44, 1.77) | -0.31 (-1.39, 0.77) |
| Four or more | 1.65 (0.25, 3.04)^c^ | -0.11 (-1.48, 1.26) | 1.65 (0.25, 3.04)^c^ | -0.14 (-1.52, 1.24) |
| Individual ACE exposure |  |  |  |  |
| Bullying | 0.30 (-0.79, 1.39) | -0.61 (-1.63, 0.40) | 0.28 (-0.82, 1.38) | -0.60 (-1.62, 0.42) |
| Emotional abuse | 1.20 (0.15, 2.26)^c^ | -0.31 (-1.34, 0.71) | 1.23 ( 0.17, 2.29)^c^ | -0.36 (-1.40, 0.68) |
| Emotional neglect | -0.45 (-1.53, 0.64) | 0.31 (-0.69, 1.30) | -0.43 (-1.52, 0.65) | 0.31 (-0.69, 1.30) |
| Parent mental health problem | 0.91 (-0.01, 1.83) | -0.21 (-1.05, 0.64) | 0.90 (-0.02, 1.83) | -0.22 (-1.07, 0.63) |
| Parent convicted | 0.56 (-1.00, 2.12) | -0.15 (-1.51, 1.21) | 0.55 (-1.01, 2.11) | -0.13 (-1.49, 1.24) |
| Parental separation | 0.90 (-0.29, 2.10) | 0.62 (-0.34, 1.58) | 0.90 (-0.30, 2.09) | 0.61 (-0.36, 1.57) |
| Physical abuse | 1.22 (0.06, 2.38)^c^ | 0.04 (-1.11, 1.19) | 1.22 (0.06, 2.38)^c^ | 0.02 (-1.14, 1.18) |
| Sexual abuse | 1.29 (-0.71, 3.30) | -0.75 (-3.67, 2.17) | 1.28 (-0.73, 3.28) | -0.79 (-3.71, 2.13) |
| Household substance abuse | 0.72 (-0.78, 2.22) | 0.43 (-0.78, 1.65) | 0.74 (-0.76, 2.24) | 0.42 (-0.79, 1.64) |
| Violence in household | -0.15 (-1.28, 0.99) | 0.72 (-0.35, 1.79) | -0.15 (-1.28, 0.98) | 0.70 (-0.37, 1.78) |

ACE, adverse childhood experience; 95% CI, 95% confidence interval

^a^ Model 1 adjusted for white blood cell composition, smoking status at time of DNA methylation measurement, maternal body mass index, maternal smoking during pregnancy, maternal age at delivery, maternal depression during pregnancy, partner’s depression during pregnancy, mother’s highest education qualification, household’s highest socioeconomic class

^b^ Model 2 adjusted for the same covariates as Model 1 with the addition of body mass index

^c^ Two-tailed *P* value < 0.05
